# A synergistic impact of body mass index and gamma gap on heart failure and mortality rate among older patients with coronary artery disease: a prospective study with 10-year follow-up

**DOI:** 10.1038/s41387-023-00255-1

**Published:** 2023-12-05

**Authors:** Xiaofei Liu, Yangrui Zheng, Da Li, Yali Zhao, Houchen Lv, Lixun Guan, Shihui Fu

**Affiliations:** 1 Department of Rheumatology and Immunology, Hainan Hospital of Chinese People’s Liberation Army General Hospital, Sanya, China; 2 Neurosurgery Department, Hainan Hospital of Chinese People’s Liberation Army General Hospital, Sanya, China; 3 State Key Laboratory of Stem Cell and Reproductive Biology, Institute of Zoology, Chinese Academy of Sciences, Beijing, China; 4 Central Laboratory, Hainan Hospital of Chinese People’s Liberation Army General Hospital, Sanya, China; 5 Orthopedics Department, Chinese People’s Liberation Army General Hospital, Beijing, China; 6 Hematology Department, Hainan Hospital of Chinese People’s Liberation Army General Hospital, Sanya, China; 7 Department of Cardiology, Hainan Hospital of Chinese People’s Liberation Army General Hospital, Sanya, China; 8 Department of Geriatric Cardiology, Chinese People’s Liberation Army General Hospital, Beijing, China

**Keywords:** Cardiovascular diseases, Geriatrics, Preventive medicine, Risk factors

## Abstract

**Purpose:**

This prospective study with 10-year follow-up aimed to analyze potential impact of body mass index (BMI) and gamma gap on heart failure and mortality rate in older patients with coronary artery disease (CAD).

**Methods:**

There were 987 consecutive older patients with CAD included and divided into four groups according to BMI and gamma gap levels.

**Results:**

Median age was 86 years. The highest proportion of heart failure (46.2%) and the highest mortality rate (84.4%) was observed in patients with low BMI and high gamma gap, whereas the lowest proportion of heart failure (18.9%) and the lowest mortality rate (62.9%) was observed in those with high BMI and low gamma gap. After full adjustment in multivariate Logistic regression analysis, heart failure was most common in patients with low BMI and high gamma gap compared with those with high BMI and low gamma gap (hazard ratio [HR]: 2.82, 95% confidence interval [CI]: 1.79–4.48, *P* < 0.05). Meanwhile, multivariate Cox regression analysis showed that mortality rate was the highest in those with low BMI and high gamma gap compared with patients with high BMI and low gamma gap (HR: 1.65, 95% CI: 1.32–2.07, *P* < 0.05).

**Conclusion:**

The combination of low BMI and high gamma gap could further promote heart failure and increase mortality rate in older patients with CAD. Future studies should explore the underlying mechanisms linking low BMI, high gamma gap, and mortality rate, as well as the potential benefits of nutritional and immunological interventions to improve health prognosis in older patients with CAD.

## Introduction

High body mass index (BMI) is considered a high-risk factor for the occurrence of cardiovascular disease (CAD) [1]. Previous research has reported that high BMI leads to a 13.5% mortality rate among patients with CAD [2]. However, other scholars have suggested that the overall mortality rate of patients with obesity with CAD is lower than that of normal-weight individuals, which is known as the obesity paradox [3], but its underlying reasons are unclear [4].

Gamma gap, also known as globulin fraction, defined as the difference between the serum total protein levels and the serum albumin levels, has been reported to be associated with all-cause mortality rate in several studies [5, 6]. However, there is no information regarding the association between gamma gap and mortality rate in different BMI categories. Mortality rate may vary according to different gamma gap and BMI categories. Therefore, in this prospective study with 10-year follow-up, we aimed to analyze potential impact of BMI and gamma gap on heart failure and mortality rate in older patients with CAD.

## Method

### Study population

This prospective study included 987 consecutive patients with CAD aged 60 years or older in Department of Geriatric Cardiology, Chinese People’s Liberation Army (PLA) General Hospital. Chinese PLA General Hospital is their designated hospital with comprehensive medical treatment and final death records, which makes it easier for us to track them effectively and judge endpoint accurately. The diagnosis of CAD was made by the chief physician based on medical history, angina symptoms, cardiac biomarkers, and specific tests, such as electrocardiogram (rest/exercise), echocardiography, nuclear imaging, computed tomography, and coronary angiography, based on the guidelines of the American College of Cardiology (ACC)/American Heart Association (AHA)/European Society of Cardiology (ESC) [7, 8]. Exclusion criteria: severe aortic stenosis, expected heart transplantation, and ventricular assist device. This study was approved by the Ethics Committee of Chinese PLA General Hospital in Beijing, China. Informed consent was obtained from participants at the time of admission, and the study followed the principles of the Helsinki Declaration of 1975.

### Baseline characteristics

The available baseline characteristics analyzed included demographics (age and gender), physical examination (height, weight, heart rate, systolic blood pressure, and diastolic blood pressure [SBP and DBP]), laboratory measurements (hemoglobin, albumin, total cholesterol, high-density lipoprotein cholesterol [HDL-C], low-density lipoprotein cholesterol [LDL-C], fasting plasma glucose [FPG], creatinine, C-reactive protein [CRP], and N-terminal pro-B-type natriuretic peptide [NT-proBNP]). BMI was calculated as body weight (kg) divided by the square of height (m). Gamma gap, also known as globulin fraction, was defined as the serum total protein levels minus the serum albumin levels [5, 6]. BMI ≥ 25 kg/m^2^ was defined as high BMI, and gamma gap ≥30 g/L was defined as high gamma gap. Data management involved experienced and trained doctors who recorded the data in a database. Other doctors performed logical checks to ensure the accuracy of the database.

### Follow-up and study endpoints

The endpoint of this study was all-cause mortality. Considering the higher incidence of multi-organ failure in older patients and the priority of all-cause mortality in prognostic studies, all-cause mortality was the predetermined endpoint of this study. Follow-up continued for almost 10 years, and no patient lost to the follow-up. Follow-up results were directly obtained from medical records and telephone interviews. Deaths were determined by legal documents of death time and place.

### Statistical analysis

Continuous variables with skewed distribution were presented as medians and interquartile ranges (IQR), while categorical variables were presented as frequencies and percentages. Kruskal-Wallis test was used to analyze potential difference of continuous variables with skewed distribution. Chi-square test was used to test potential difference of categorical variables. Kaplan-Meier curve was used to describe cumulative survival of patients with different BMI or gamma gap levels. Multivariate Logistic regression analysis was used to examine potential impact of BMI and gamma gap on heart failure. Multivariate Cox regression analysis was used to examine potential impact of BMI and gamma gap on mortality rate. All analyses were performed using IBM SPSS 22.0 software. A *P* value of <0.05 was considered significant.

## Result

### Characteristic description of patients with different BMI or gamma gap levels

Median age of 987 patients was 86 years (IQR: 82–90 years). Patients with high BMI accounted for 41.9% (414 patients), and those with high gamma gap accounted for 31.8% (314 patients). The average follow-up period of this study was 1836 days (median: 1871 days [IQR: 384–3225 days]). Age, gender, BMI, heart rate, DBP, hemoglobin, albumin, HDL-C, FPG, creatinine, CRP, NT-proBNP, and gamma gap had significant distinction in patients with different BMI or gamma gap levels (all *P* < 0.05, Table 1). The highest proportion of heart failure (46.2%) and the highest mortality rate (84.4%) was observed in patients with low BMI and high gamma gap, whereas the lowest proportion of heart failure (18.9%) and the lowest mortality rate (62.9%) was observed in those with high BMI and low gamma gap.

**Table 1:** Baseline characteristics of patients with different BMI and gamma gap levels.

Characteristics	All patients (*n* = 987)	High BMI, low gamma gap (*n* = 286)	Low BMI, low gamma gap (*n* = 387)	High BMI, high gamma gap (*n* = 128)	Low BMI, high gamma gap (*n* = 186)	*P* value
Age, yrs	86.0 (82.0–90.0)	86.0 (81.0–89.0)	86.0 (82.0–90.0)	85.0 (82.0–90.0)	87.0 (83.0–90.0)	0.046
Males (%)	887 (89.9)	268 (93.7)	347 (89.7)	108 (84.4)	164 (88.2)	0.024
BMI,kg/m^2^	24.2 (21.7–26.6)	26.9 (25.9–29.1)	22.2 (20.5–23.7)	27.4 (26.0–29.7)	22.2 (20.2–23.6)	<0.001
Smoking history(%)	371 (37.6)	105 (36.7)	156 (40.3)	44 (34.4)	66 (35.5)	0.526
Heart rate, beats/min	72.0 (64.0–80.0)	70.0 (64.0–78.0)	72.0 (64.0–80.0)	73 (67.0–82.0)	75.0 (66.0–85.0)	0.001
SBP, mmHg	132.5 (124.0–142.0)	135.0 (126.0–142.5)	131.8 (123.0–141.4)	133.2 (125.1–143.0)	131.1 (122.0–141.2)	0.098
DBP, mmHg	69.0 (63.7–74.3)	70.0 (64.8–76.1)	68.3 (63.7–73.0)	69.6 (63.4–76.2)	68.7 (63.0–73.4)	0.005
Hemoglobin, g/L	124.0 (108.0–137.0)	131.0 (116.0–143.0)	123.0 (108.0–134.0)	119.5 (106.5–135.0)	115.0 (99.8–132.0)	<0.001
Albumin,g/L	38.2 (35.2–40.7)	39.0 (36.7–41.1)	38.5 (35.6–40.8)	37.7 (35.0–40.5)	36.0 (33.0–39.9)	<0.001
TC, mmol/L	3.8 (3.3–4.4)	3.8 (3.3–4.2)	3.8 (3.2–4.4)	3.8 (3.3–4.3)	3.9 (3.3–4.6)	0.133
HDL-C,mmol/L	1.1 (0.9–1.8)	1.0 (0.9–1.2)	1.1 (1.0–1.4)	1.0 (0.9–1.1)	1.0 (0.8–1.2)	<0.001
LDL-C, mmol/L	2.1 (1.7–2.6)	2.2 (1.7–2.6)	2.1 (1.6–2.6)	2.2 (1.7–2.6)	2.2 (1.7–2.9)	0.197
FPG, mmol/L	5.38 (4.83–6.14)	5.4 (4.9–6.0)	5.2 (4.7–5.9)	5.7 (5.0–6.7)	5.5 (4.8–6.4)	<0.001
Creatinine, μmol/L	89.0 (7.0–117.0)	89.0 (74.8–110.3)	86.0 (70.9–111.9)	94.6 (80.0–123.9)	100.9 (75.0–150.5)	0.001
CRP	0.6 (0.2–2.5)	0.4 (0.1–1.5)	0.4 (0.1–1.7)	0.9 (0.4–2.8)	1.7 (0.5–5.2)	<0.001
NT-proBNP, pg/ml	423.4 (169.3–1855.2)	266.1 (116.8–972.8)	418.2 (176.5–1756.0)	664.7 (182.0–1900.3)	996.4 (258.5–3852.6)	<0.001
Gamma gap,g/L	27.4 (24.2–31.0)	25.2 (22.8–27.1)	25.7 (23.2–27.7)	32.7 (31.0–35.6)	33.3 (31.3–36.1)	<0.001
Heart failure (%)	295 (29.9)	54 (18.9%)	114 (29.5%)	41 (32.0%)	86 (46.2%)	<0.001
Mortality rate (%)	717 (72.6)	180 (62.9%)	274 (70.8%)	106 (82.8%)	157 (84.4%)	<0.001

Data are presented as median (interquartile range) or *n* (%).

*BMI* body mass index, *SBP* systolic blood pressure, *DBP* diastolic blood pressure, *TC* total cholesterol, *HDL-C* high density lipoprotein cholesterol, *LDL-C* low density lipoprotein cholesterol, *FPG* fasting plasma glucose, *CRP* C-reactive protein, *NT-proBNP* N-terminal pro-brain natriuretic peptide.

### A synergistic impact of BMI and gamma gap on heart failure

High BMI was negatively correlated with heart failure (r: -0.13, *P* < 0.001). Proportion of heart failure was significantly higher in patients with low BMI than that in those with high BMI (34.9% vs 22.9%, *P* < 0.001). High gamma gap was positively correlated with heart failure (r: 0.16, *P* < 0.001). Proportion of heart failure was significantly higher in patients with high gamma gap than that in those with low gamma gap (40.4% vs 25.0%, *P* < 0.001). Multivariate Logistic regression analysis showed that compared with patients with low BMI, those with high BMI had a significantly lower proportion of heart failure (hazard ratio [HR]: 0.56, 95% confidence interval [CI]: 0.42–0.74, *P* < 0.001). Compared with patients with low gamma gap, those with high gamma gap had a significantly higher proportion of heart failure (HR: 2.04, 95% CI: 1.54–2.72, *P* < 0.001).

Compared with patients with high BMI and low gamma gap, those with low BMI and low gamma gap (HR: 1.79, 95% Cl: 1.24–2.59) or those with high BMI and high gamma gap had significantly higher proportions of heart failure (HR: 2.03, 95% CI: 1.26–3.26), and those with low BMI and high gamma gap had the highest proportion of heart failure (HR: 3.70, 95% CI: 2.44–5.59, all *P* < 0.05, Table 2). After adjusting for age and gender, the results showed that compared with patients with high BMI and low gamma gap, heart failure was significantly more common in those with low BMI and low gamma gap (HR: 1.76, 95% CI: 1.20–2.56) or in those with high BMI and high gamma gap (HR: 1.82, 95% CI: 1.11–2.99), and most common in those with low BMI and high gamma gap (HR: 3.76, 95% CI: 2.45–5.77, all *P* < 0.05). After adjusting for all factors with significant distinction shown in Table 1, compared with patients with high BMI and low gamma gap, heart failure was more common in those with low BMI and low gamma gap (HR: 1.62, 95% CI: 1.08–2.42), and most common in those with low BMI and high gamma gap (HR: 2.82, 95% CI: 1.79–4.48, all *P* < 0.05).

**Table 2:** A synergistic impact of BMI and gamma gap on heart failure with different adjustment models.

Heart failure	Hazard ratio (95% confidence interval), *P* value			
	High BMI, low gamma gap (*n* = 286)	Low BMI, low gamma gap (*n* = 387)	High BMI, high gamma gap (*n* = 128)	Low BMI, high gamma gap (*n* = 186)
Model 1	Reference	1.79 (1.24–2.59), *P* = 0.002	2.03 (1.26–3.26), *P* = 0.004	3.70 (2.44–5.59), *P* <0.001
Model 2	Reference	1.76 (1.20–2.56), *P* = 0.004	1.82 (1.11–2.99), *P* = 0.017	3.76 (2.45–5.77), *P* <0.001
Model 3	Reference	1.62 (1.08–2.42), *P* = 0.019	1.58 (0.94–2.65), *P* = 0.082	2.82 (1.79–4.48), *P* <0.001

Model 1: unadjusted; model 2: adjusted for age and sex; model 3: adjusted for age, gender, heart rate, diastolic blood pressure, hemoglobin, albumin, high density lipoprotein cholesterol, creatinine, fasting plasma glucose, C-reactive protein and N-terminal pro-brain natriuretic peptide.

*BMI* body mass index.

### A synergistic impact of BMI and gamma gap on mortality rate

**Fig. 1:**
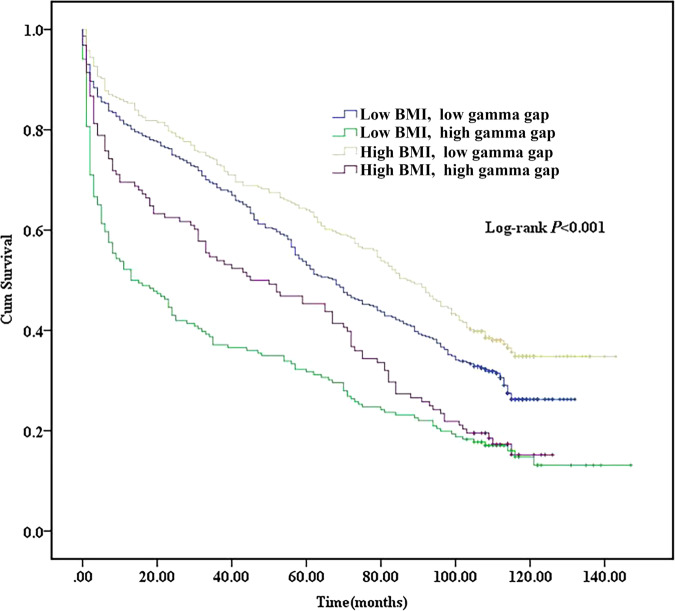
Kaplan-Meier analysis of survival rate according to body mass index and gamma gap levels. There was significant difference in survival rate between different groups (*P* < 0.001).

High BMI was negatively correlated with mortality rate (r: -0.07, *P* < 0.05). Mortality rate was significantly higher in patients with low BMI than in those with high BMI (75.2% vs 69.1%, *P* < 0.05). High gamma gap was positively correlated with mortality rate (r: 0.17, *P* < 0.001). Mortality rate was significantly higher in patients with high gamma gap than in those with low gamma gap (83.8% vs 67.5%, *P* < 0.001). Kaplan-Meier analysis showed significant difference in survival rate between different groups (*P* < 0.001, Fig. 1). Multivariate Cox regression analysis showed that compared with patients with low BMI, those with high BMI had a significantly lower mortality rate (HR: 0.80, 95% CI: 0.69–0.93, *P* < 0.05). Compared with patients with low gamma gap, those with high gamma gap had a significantly higher mortality rate (HR: 1.81, 95% CI: 1.55–2.10, *P* < 0.001).

Compared with patients with high BMI and low gamma gap, those with low gamma gap and low BMI (HR:1.25, 95% CI: 1.04–1.51) or those with high BMI and high gamma gap (HR: 1.78, 95% CI: 1.40–2.27) had higher mortality rates, and those with low BMI and high gamma gap had the highest mortality rate (HR: 2.30, 95% CI: 1.85–2.85, all *P* < 0.05, Table 3). After adjusting for age and gender, the results showed that compared with patients with high BMI and low gamma gap, mortality rate was significantly higher in those with high BMI and high gamma gap (HR: 1.68, 95% CI: 1.32–2.14), and the highest in those with low BMI and high gamma gap (HR: 2.20, 95% CI: 1.78–2.74, all *P* < 0.001). After adjusting for all factors with significant distinction shown in Table 1, compared with patients with high BMI and low gamma gap, mortality rate was significantly higher in those with high BMI and high gamma gap (HR: 1.51, 95% CI: 1.19–1.93), and the highest in those with low BMI and high gamma gap (HR: 1.65, 95% CI: 1.32–2.07, all *P* < 0.05).

**Table 3:** A synergistic impact of BMI and gamma gap on mortality rate with different adjustment models.

Mortality rate	Hazard ratio (95% confidence interval), *P* value			
	High BMI, low gamma gap (*n* = 286)	Low BMI, low gamma gap (*n* = 387)	High BMI, high gamma gap (*n* = 128)	Low BMI, high gamma gap (*n* = 186)
Model 1	Reference	1.25 (1.04–1.51), *P* = 0.019	1.78 (1.40–2.27), *P* <0.001	2.30 (1.85–2.85), *P* <0.001
Model 2	Reference	1.19 (0.98–1.43), *P* = 0.075	1.68 (1.32–2.14), *P* <0.001	2.20 (1.78–2.74), *P* <0.001
Model 3	Reference	1.09 (0.90–1.33), *P* = 0.380	1.51 (1.19–1.93), *P* = 0.001	1.65 (1.32–2.07), *P* <0.001

Model 1: unadjusted; model 2: adjusted for age and sex; model 3: adjusted for age, gender, heart rate, diastolic blood pressure, hemoglobin, albumin, high density lipoprotein cholesterol, creatinine, fasting plasma glucose, C-reactive protein and N-terminal pro-brain natriuretic peptide.

*BMI* body mass index.

## Discussion

In this prospective study with 10-year follow-up, both low BMI and high gamma gap contribute to heart failure and mortality rate in older patients with CAD, suggesting that low BMI and high gamma gap play a synergistic impact on heart failure and mortality rate.

In this study, we found that low BMI and high gamma gap were associated with an increased risk of mortality rate, even after adjusting for age, gender, and other potential confounding factors. The association between BMI and mortality rate is inconsistent, which is considered as obesity paradox [9, 10]. Some studies have suggested that overweight was an important risk factor for the development of heart failure and mortality rate [11, 12], but other researches realized that high BMI had a protective impact on the actual long-term prognosis in patients with CAD [13–16]. Our findings suggested that high BMI is negatively associated with the development of heart failure and mortality rate in older patients with CAD.

The association between gamma gap and mortality rate in older patients with CAD has not been widely studied, not to say the underlying mechanisms. In this study, we found that high gamma gap resulted in increased heart failure and mortality rate in older patients with CAD. Elevated gamma gap has been realized to increase mortality rate in populations of nonagenarians and centenarians [17, 18]. High gamma gap suggests the presence of systemic inflammation and immune dysfunction [19, 20], which are important factors contributing to the development of atherosclerosis [21, 22]. An elevated gamma gap may result from increased globulin levels or decreased albumin levels. Decreased albumin levels have been shown to be a risk factor for all-cause mortality. Studies have indicated that decreased albumin levels are associated with higher mortality rate in octogenarians [23] and can predict long-term mortality rate in patients with dual-chamber permanent pacemakers [24]. Immune activation and pro-inflammatory cytokines play important roles in the progression of heart failure [25, 26], and elevated circulating globulin levels are a predictor of heart failure and mortality rate [27, 28].

Our finding of a synergistic impact of low BMI and high gamma gap on heart failure and mortality risk is still novel and warrants further investigation. One possible explanation is that low BMI and high gamma gap may reflect a state of chronic malnutrition and inflammation, which could lead to a higher risk of adverse prognosis. There is another potential mechanism may explain the relationship between BMI and gamma gap. The large number of activated macrophages in the adipose tissue increases the catabolism of globulin [29]. The half-life of globulin in individuals with obesity is shorter compared with lean individuals [30].

## Conclusion

This prospective study with 10-year follow-up provided new evidence that the combination of low BMI and high gamma gap could further promote heart failure and increase mortality rate in older patients with CAD. Future studies should explore the underlying mechanisms linking low BMI, high gamma gap, and mortality rate, as well as the potential benefits of nutritional and immunological interventions to improve health prognosis in older patients with CAD.

## Data Availability

All data and material are available under the requirement to the corresponding authors.
